# Bio.Phylo: A unified toolkit for processing, analyzing and visualizing phylogenetic trees in Biopython

**DOI:** 10.1186/1471-2105-13-209

**Published:** 2012-08-21

**Authors:** Eric Talevich, Brandon M Invergo, Peter JA Cock, Brad A Chapman

**Affiliations:** 1 Institute of Bioinformatics, University of Georgia, 120 Green Street, Athens, GA 30602, USA; 2 Institute of Evolutionary Biology (CSIC-UPF), CEXS-UPF-PRBB, C/ Doctor Aiguader 88, 08003 Barcelona, Spain; 3 James Hutton Institute, InvergowrieDundee DD2 5DA, UK; 4 Harvard School of Public Health Bioinformatics Core, 655 Huntington Ave, Boston, MA 02115, USA

## Abstract

**Background:**

Ongoing innovation in phylogenetics and evolutionary biology has been accompanied by a proliferation of software tools, data formats, analytical techniques and web servers. This brings with it the challenge of integrating phylogenetic and other related biological data found in a wide variety of formats, and underlines the need for reusable software that can read, manipulate and transform this information into the various forms required to build computational pipelines.

**Results:**

We built a Python software library for working with phylogenetic data that is tightly integrated with Biopython, a broad-ranging toolkit for computational biology. Our library, Bio.Phylo, is highly interoperable with existing libraries, tools and standards, and is capable of parsing common file formats for phylogenetic trees, performing basic transformations and manipulations, attaching rich annotations, and visualizing trees. We unified the modules for working with the standard file formats Newick, NEXUS and phyloXML behind a consistent and simple API, providing a common set of functionality independent of the data source.

**Conclusions:**

Bio.Phylo meets a growing need in bioinformatics for working with heterogeneous types of phylogenetic data. By supporting interoperability with multiple file formats and leveraging existing Biopython features, this library simplifies the construction of phylogenetic workflows. We also provide examples of the benefits of building a community around a shared open-source project. Bio.Phylo is included with Biopython, available through the Biopython website, http://biopython.org.

## Background

Comparative methods in biology have been an active area of scientific software development since the wider availability of computing resources made such large-scale quantitative analyses feasible [1]. In recent years, the number and range of tools for working with phylogenetic information alone has expanded dramatically, creating new opportunities as well as challenges in data integration [2]. At the same time, we have seen new efforts to create standards for data exchange and storage [3-6]. There is thus a growing need for modular software toolkits that can integrate cleanly into workflows for bioinformatics analyses that include phylogenetic data, as well as facilitate tool development by providing a high-level application programming interface (API) independent of data storage formats.

Freely available, open-source software libraries now play a major role in integrating software components for bioinformatics analyses. Generalized toolkits for working with phylogenetic information are already available and in wide use, including the Ape package for the R statistical programming language [7], BioPerl [8] and Bio::Phylo [2] for Perl, and Mesquite [9] for Java. The Open Bioinformatics Foundation in particular supports an ecosystem of broadly aimed bioinformatics libraries, collectively referred to as the Bio* or “Bio-star” projects. These include BioPerl [8], Biopython [10], BioJava [11], BioRuby [12], BioLib [13] and BioSQL [14]. All of these libraries provide common functionality to each of their target programming languages or environments: read and write a variety of file formats used in bioinformatics, communicate with public web services such as NCBI Entrez [15], access widely used stand-alone programs such as BLAST [16,17], and define fundamental data types for storing biological information, such as annotated sequence records, protein structures and phylogenetic trees.

We chose Python as the implementation language for our library due to its increasing usage in scientific work [18]. It allows for particularly concise, easy-to-read code, has extensive library support, and enables the same code to be run on all major operating systems. In addition to Biopython, several other high-quality Python libraries are available for phylogenetics: PyCogent [19], DendroPy [20], ETE [21] and p4 [22] each serve specific problem domains well. However, they focus specificially on phylogenetics and are not intended to be general-purpose frameworks for processing biological data.

In this article we present a framework for phylogenetics in the Python programming language, fully integrated with the Biopython toolkit. We supplemented existing support for the NEXUS [23] and Newick [24] standards in Biopython with a full-featured phylogenetic module, Bio.Phylo, incorporating new input/output support for the phyloXML standard [5]. We then integrated the new I/O infrastructure with related code in Biopython and added more features that would be useful for researchers, with a focus on easy interoperability and rapid script development.

Our contributions include an I/O framework with parsers and serializers for the standard Newick, NEXUS and phyloXML formats for phylogenetic trees, integrated through a consistent and simple API that is familiar to users of Biopython and BioPerl, as well as a set of common functions to perform on trees independently of the source data format. This framework includes comprehensive class definitions for the rich annotation types which can be serialized in the standard phyloXML format. We also provide several options for visualizing trees, and convenient integration with popular third-party tools.

## Implementation

Bio.Phylo is written as a sub-package within the Biopython code base, and is installed along with the Biopython distribution. It has been available as part of the Biopython library since version 1.54, released in May 2010.

The library can be used in Python versions 2.5 through 2.7 and 3.1 or later, without any external libraries required at the time of installation. Functions that rely on external libraries are written within a separate module of the code base, and import their dependencies at run-time. This design makes it possible to install Biopython and use the rest of Bio.Phylo without having installed the dependencies for these specific functions. Because Bio.Phylo is written entirely in Python, it also runs on alternative implementations of Python: Jython 2.5 and Pypy 1.6 through 1.9 in particular pass the module’s unit testing suite.

### I/O functions for standard file formats

A unified API for input and output is provided for the Newick, NEXUS and phyloXML formats with the same underlying object structures. The simple API style is shared with Bio.SeqIO, AlignIO, Motif and other modules within Biopython; the API also resembles that of BioPerl. The read and write functions accept a filename or handle, so they also work with Unix pipes, network handles or other file-like objects. As in Biopython’s SeqIO and AlignIO, a convert function is available to convert between any two of the supported formats with a single call.

The I/O code is designed for simple addition of other file formats without disturbing the existing code for Newick, NEXUS and phyloXML. Parsing and serialization code is separated from the internal tree object representation. All parsers return a common object type, the tree, independently of the source data format, and the parsed tree objects all support a common set of operations.

### Common tree representation

A phylogeny is represented by a Tree object which contains global information about the phylogeny, such as whether it is rooted, and a reference to the root Clade. Each Clade contains a reference to its child clades, a simple Python list of further Clade objects, nested recursively. The Clade object also contains information about the node occuring at the split or tip it represents, such as the length of the branch leading to it and the name of the node. There is no additional complex data structure operating “under the hood”, and trees are not required to be bifurcating, although the functions in Bio.Phylo currently assume that each Clade has a single parent, i.e., the topology of the Tree is indeed a tree and not a network. This straightforward design is conducive to implementing algorithms in a form that is easy to read and understand, with minimal need for management of the object’s internal data representation.

The basic Tree and Clade objects store the intersection of the information that can be represented in the Newick and phyloXML formats. To store additional format-specific attributes, we defined separate Newick and PhyloXML sub-classes which inherit from the basic Tree and Clade classes. Classes for each element type defined in the phyloXML specification have been implemented in the Bio.Phylo.PhyloXML module, allowing richer annotation types to be attached to Tree objects. For convenience in adding graphical cues, the Clade class also has attributes for the displayed color and width of a branch. The properties of the branch color and width attributes follow the phyloXML specification, and are available on the common Clade class due to their usefulness, particularly during interactive work. In accordance with the phyloXML specification, these attributes apply in a cascading manner down the clade: for example, if the root Clade object is assigned the color blue, the entire tree will be displayed as blue unless a child clade overrides this attribute.

### Methods for tree inspection and manipulation

The Tree and Clade objects also implement common methods for tree manipulation and simple analyses that might be used routinely in bioinformatics workflows. These include methods for tree search and traversal, extracting basic information, and modifying or manipulating the tree. A listing of these methods and other functions available in Bio.Phylo is given in Table 1.

**Table 1:** Built-in functions and tree methods

**Source**	**Function**	**Description**
Bio.Phylo	read	Parse a file in the given format and return a single tree.
Bio.Phylo	parse	Iteratively parse a file and return each of the trees it contains.
Bio.Phylo	write	Write a sequence of trees to file in the given format.
Bio.Phylo	convert	Convert between two tree file formats.
Bio.Phylo	draw	Plot the given tree using matplotlib (or pylab).
Bio.Phylo	draw_ascii	Draw an ascii-art phylogram of the given tree.
Bio.Phylo	draw_graphviz	Display a tree or clade as a graph, using the graphviz engine.
Bio.Phylo	to_networkx	Convert a Tree object to a NetworkX graph object.
*Bio.Phylo.BaseTree*		
TreeMixin	find_elements	Find all tree elements matching the given attributes.
TreeMixin	find_clades	Find each clade containing a matching element.
TreeMixin	find_any	Return the first matching element found by find_elements, if any.
TreeMixin	get_path	List the clades directly between the current node and the target.
TreeMixin	get_nonterminals	List of all of the tree or clade’s internal nodes.
TreeMixin	get_terminals	List of all of the tree or clade’s “leaf” nodes.
TreeMixin	trace	List of all clade object between two targets in the tree/clade.
TreeMixin	common_ancestor	Most recent common ancestor (clade) of all the given targets.
TreeMixin	count_terminals	Count the number of terminal nodes within the tree.
TreeMixin	depths	Create a mapping of tree clades to depths (by branch length).
TreeMixin	distance	Calculate the sum of the branch lengths between two targets.
TreeMixin	is_bifurcating	Return True if tree downstream of node is strictly bifurcating.
TreeMixin	is_monophyletic	If the given terminals comprise a complete subclade, return the MRCA.
TreeMixin	is_parent_of	True if target is a descendent of the tree.
TreeMixin	is_preterminal	True if all direct descendents are terminal.
TreeMixin	total_branch_length	Calculate the sum of all the branch lengths in the tree.
TreeMixin	collapse	Deletes target from the tree, relinking its children to its parent.
TreeMixin	collapse_all	Collapse all the descendents of the tree, leaving only terminals.
TreeMixin	ladderize	Sort clades in-place according to the number of terminal nodes.
TreeMixin	prune	Prunes a terminal clade from the tree.
TreeMixin	split	Generate *n* (default 2) new descendants.
Tree, Clade	is_terminal	True if the node has no descendents.
Tree	root_with_outgroup	Reroot the tree with the specified outgroup clade.
Tree	root_at_midpoint	Reroot the tree at the midpoint between the two most distant terminals.
Tree	format	Serialize the tree as a string in the specified file format.
Tree	as_phyloxml	Convert the tree to its PhyloXML subclass equivalent.
Tree	from_clade	Create a new Tree object given a clade.
Tree	randomized	Create a randomized bifurcating tree, given a list of taxa.
*Bio.Phylo.PhyloXML*		
Phylogeny	to_alignment	Construct an alignment from the aligned sequences in this tree.
*Bio.Phylo.PAML*		
chi2	cdf_chi2	*χ*^2^ cumulative distribution function, for log-likelihood ratio tests.
baseml	read	Parse a BASEML results file.
codeml	read	Parse a CODEML results file.
yn00	read	Parse a yn00 results file.
_paml.Paml	write_ctl_file	Dynamically build a program-specific control file.
_paml.Paml	read_ctl_file	Parse a control file to create a program-specific class instance.
_paml.Paml	print_options	Print all of the program options and their current settings.
_paml.Paml	set_options	Set the value of a program option.
_paml.Paml	get_option	Return the value of a program option.
_paml.Paml	get_all_options	Return the current values of all the program options.
_paml.Paml	run	Run a PAML program and parse the results.

Public methods and functions provided in the Bio.Phylo module and sub-modules. An up-to-date version of this information is available at http://biopython.org/DIST/docs/api/.

Both the Tree and Clade classes inherit from a third class, TreeMixin, which implements these common tree methods. Therefore, most of the same methods are available on both the Tree object and any of the Clade objects it contains; in practice, one can usually ignore the distinction between the global Tree object and the root Clade.

### Visualization

**Figure 1:**
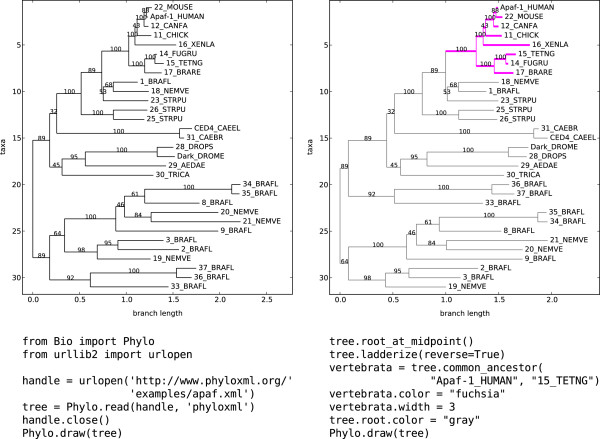
**Tree visualization.** An example tree with the code to generate each plot shown below each plot. First, a phyloXML tree of the Apaf-1 protein family [27] is downloaded, read by Bio.Phylo, and plotted with default settings. The tree is then rerooted at the midpoint of its two most divergent tips and ladderized such that sibling clades with a larger number of descendents are listed first. The clade of genes belonging to vertebrate species is identified as the common ancestor of the human and zebrafish, after inspection of the original tree. The vertebrate clade is highlighted with the color fuchsia and an increased branch width, and the rest of the tree is colored gray. Finally, the tree is plotted again

We implemented several mechanisms for displaying trees. The draw function displays a rooted phylogram (Figure 1), in the style of Phylip’s drawgram program, while draw_graphviz displays an unrooted cladogram using the Graphviz programs [25] for layout and the NetworkX library [26] as an intermediate graph representation.

The generated plots can be further modified using the built-in functions of matplotlib or PyLab [28]. In case no visualization libraries are available on a system, plain-text representations of a tree are also possible. The function draw_ascii draws an “ASCII-art” tree to standard output or a given file handle. Using the built-in Python print statement on a Tree object shows the nested object hierarchy, including class names and the values of attributes such as branch length.

Since the complete phyloXML specification is implemented, files can be saved by Bio.Phylo with graphical annotations and then rendered with popular visualization tools such as Archaeopteryx [29]. The cascading behavior of clade colors and branch widths is shared by Archaeopteryx and other tree visualization software that implements phyloXML.

### Wrappers for third-party programs

Biopython includes a common framework for building wrappers for external programs. This framework allows us to leverage the functionality of widely used third-party programs from within Biopython, rather than reimplement those functions. For example, Biopython supports the Phylip suite of programs via EMBOSS [30], with wrappers implemented in the Bio.Emboss.Applications module. We follow this rationale in Bio.Phylo.

Within the sub-module Bio.Phylo.Applications, we currently provide wrappers for the tree inference programs PhyML [31] and RAxML [32]. The code scaffolding is in place to add more, using the same common Bio.Application framework.

#### PAML integration

Bio.Phylo also includes wrappers for the PAML suite of programs [33], specifically the analysis programs yn00, baseml, and codeml. Additionally, we created a pure-Python re-implementation of the program chi2 as a simple means to perform likelihood ratio tests. Since the analysis programs are operated through the use of configuration files rather than command-line arguments, a different approach was required than what Bio.Application enables. We therefore provide wrappers for these programs in a separate sub-module, Bio.Phylo.PAML.

For each of the three analysis programs, the wrapper defines a corresponding class that the user instantiates to store the configuration state. The programs’ standard configuration options are set through the set_options method and are automatically written to a configuration file when the run method is invoked or through the write_ctl_file method. Existing configuration files may be parsed via the method read_ctl_file. Finally, each module of Bio.Phylo.PAML provides a read method, which parses the output files of the respective programs. The results of an analysis are stored hierarchically in a set of nested dictionary objects, allowing quick access by keywords.

### Biopython integration

Bio.Phylo integrates cleanly with other parts of the Biopython toolkit. We reuse existing Biopython API conventions, including classes for exceptions and warnings, as well as the packaging and installation mechanisms and the testing framework. As mentioned above, the wrappers for running external programs use a common Biopython framework, Bio.Application. NEXUS and Newick support were obtained through a refactoring of the Bio.Nexus module, originally written by Frank Kauff and Cymon J. Cox [34].

Where appropriate, classes in the PhyloXML module support methods for conversion to and from instances of the general-purpose Biopython classes for molecular sequences, annotated sequence regions and multiple sequence alignments. For example, phyloXML defines a ProteinDomain element type which represents a functional domain within a protein sequence that appears in the tree; our implementation of the ProteinDomain class includes a to_seqfeature method to convert a ProteinDomain instance to an instance of the Biopython class SeqFeature, a generalized representation of an annotated sequence region. These SeqFeature objects could then be used with the Biopython’s GenomeDiagram module [35] to create a diagram of the protein domain architecture of each sequence appearing in the phyloXML tree. The ProteinDomain class also provides a complementary method, from_seqfeature, which could be used to add domain architecture annotations to the sequences in a phyloXML record, given the corresponding GenBank files.

### Validation

A complete suite of unit tests to verify correct functioning of each module is included with the Biopython distribution. This includes the round-trip parsing and serialization of example files in each supported file format; conversion between formats; proper construction, behavior and serialization of all phyloXML element types; methods for tree inspection, traversal and manipulation; and succesful loading of optional third-party libraries, if available.

## Results

### Use of Bio.Phylo in published studies

Since the Bio.Phylo module has been made available to the community throughout its development, researchers have had the opportunity to use it in studies that have since been published.

A recent study of microbial phylogenomics used Bio.Phylo for large-scale processing of microbial gene trees, generating permutations of tree topologies by rerooting over 100,000 gene trees at each internal and external node [36]. Additional file 1 shows a simplified example of how such rerooting could be performed.

In another study of the eukaryotic protein kinase superfamily in the protozoan phylum Apicomplexa, Bio.Phylo was used to identify putative lineage-specific ortholog groups by selecting clades with significant bootstrap support from gene trees [37]. The corresponding sequences were then automatically extracted from a matching FASTA file, using Biopython’s SeqIO module, for further analysis in a computational pipeline.

### Performance

Despite the stark simplification of the underlying data structures, the majority of the built-in tree methods have a run time that is theoretically linear or constant in proportion to the number of nodes in the tree. The tree traversal methods find_clades and find_elements are Python generator functions which evaluate and emit values incrementally, as needed by the caller; this “lazy evaluation” approach uses computer memory more efficiently and avoids performing more computation than necessary; for example, the method find_any uses this property to stop traversal after finding the first element matching the user’s query and thus avoid traversing the remainder of the tree structure or evaluating any further comparisons.

We timed several benchmark operations on large tree files to evaluate the speed of Bio.Phylo on several different tasks (Table 2; Additional file 2). On more modestly sized trees and input files, these operations typically complete in a small fraction of a second.

**Table 2:** Performance

**Task**	**Input tree**	**Python 2.7**	**Python 3.2**	**PyPy 1.9**
Read a very large Newick tree	Smith 2011 angiosperm supertree			
	(55473 terminal nodes) [38]	17.45	16.85	1.214
Read the same large tree in phyloXML	Smith 2011, converted to phyloXML with			
	phylo_converter (http://phylosoft.org/)	3.805	4.318	3.937
Write the same large tree as Newick	Smith 2011	0.5238	0.7704	0.4378
Write the same large tree as phyloXML	Smith 2011	10.39	10.85	24.17
Read a medium-sized Newick tree	Davies 2004 angiosperm supertree			
	(440 terminal nodes) [39]	0.1097	0.1087	0.007312
Parse many Newick trees	Davies 2004, copies rerooted at			
	each node (816 trees)	84.91	84.29	6.812
Reroot at each node	Davies 2004	1.347	1.167	0.3450
Collapse all splits with bootstrap values less than 50	Davies 2004	2.266	2.312	2.411
Total branch length	Davies 2004	0.01322	0.01310	0.01448
Ladderize the tree	Davies 2004	0.1274	0.1190	0.1127
Count terminal nodes	Davies 2004	0.006838	0.006323	0.005914

Performance of Bio.Phylo functions and tree methods under different Python versions on several benchmark tasks. Reported execution times are the median of 101 replications of each task, in seconds (Additional file 2). Benchmarks were evaluated with Python versions 2.7.3 and 3.2.3 and PyPy version 1.9 on an Intel Xeon E5405 2.00 GHz processor with 8 GB memory, running under 64-bit Ubuntu Linux 12.04 with Biopython 1.60 installed.

### Cookbook and additional documentation

In keeping the Bio.Phylo module general-purpose and simple to begin using, we have chosen not to include niche functions, or approaches that are still the topic of active research, in the Biopython distribution. However, we nonetheless anticipate that other users of the Bio.Phylo module will want to use these features, and there is a benefit to sharing this code. We resolve this by maintaining an online “cookbook” on the Biopython wiki (http://biopython.org/wiki/Phylo_cookbook). This cookbook contains working code samples for common usage patterns, as well as exporting to object types of other libraries. In particular, we provide functions to convert a Bio.Phylo tree object to a distance or adjacency matrix using the NumPy module for Python, and to export a Bio.Phylo tree to the native tree objects used by the R package Ape [7], via the Rpy2 module, and the Python package PyCogent [19].

The main Biopython tutorial, included with the Biopython distribution and available online at http://biopython.org/, contains a chapter on the Bio.Phylo module describing its use in detail.

## Discussion

Bio.Phylo organizes phylogenetic trees as a primary data type, filling a previously underserved area of data handling within Biopython. The module directly implements tree parsing and serialization in three standard formats, as well as navigation, visualization and manipulation of phylogenetic trees, and conversion of tree data to other data types. Other important aspects of phylogenetics, including phylogenetic tree reconstruction and analysis of rate variation and ancestral character states, exist as optimized stand-alone programs by other authors; RAxML and PAML are examples of such programs that are already well accepted by the phylogenetics community. Rather than re-implement the functionality of these established third-party programs ourselves, we have opted to provide wrappers for these, and focus on providing “glue” utilities to ease the process of assembling computational workflows that involve phylogenetic data.

By building our work into an existing, popular library, we were able to take advantage of both the software infrastructure and the community of developers and users associated with Biopython. By reusing core objects and maintaining common API conventions for file parsing, the resulting software package has a familiar feel to new users who have prior experience with Biopython or BioPerl. To ensure code correctness and minimize errors introduced during additional development, we reuse the existing test framework and packaging mechanisms in Biopython. A continuous integration server http://testing.open-bio.org/biopython/ runs an automated test suite nightly on all supported Python versions, operating systems and implementations, including the Jython Java-based port of Python.

Our work also provides an example of the short- and long-term benefits of building a community of developers and users around a shared open-source project. By integrating with Biopython from the beginning, we gained access to an existing community of developers and users who have continually tested the software under a variety of environments and use cases, reported bugs, requested new features, and provided new code. A notable example of a contribution from the Biopython user community is Bio.Phylo.PAML, which began as an independent project, pypaml. We successfully integrated the pypaml source code into Biopython, with further enhancements based on feedback from Biopython developers and users.

The permissive open-source license that governs both Biopython and Bio.Phylo allows this code to be reused freely in other software, which could help overcome the pervasive problem of incompatible software support for widely used file formats.

### Future development

In future releases we intend to provide support for another recently standardized XML-based format, NeXML, an XML-based successor to NEXUS [6]. Since the core classes for phylogenies have already been defined and implemented in Bio.Phylo, the implementation of NeXML I/O is expected to be straightforward.

Another area for future growth is the addition of wrappers for other third-party applications in Bio.Phylo.Applications. Using the existing Bio.Application framework, it is straightforward to add other tools that can be run with a standard command-line interface. Other widely used programs that require specialized input, notably MrBayes and PhyloBayes, may be implemented with an approach similar to Bio.Phylo.PAML, providing a module containing both the functions to generate the configuration file and to run the program itself with the generated configuration.

Smaller features may appear first on the Cookbook wiki page, implemented as stand-alone functions, and may later be added to the official Biopython distribution.

## Conclusions

Bio.Phylo meets a growing need in bioinformatics for working with heterogeneous types of biological and phylogenetic data. By supporting interoperability with multiple file formats and leveraging existing Biopython features, this library simplifies the construction of phylogenetic workflows and computational pipelines, addressing practical issues of data integration and exchange in the bioinformatics community.

## Availability and requirements

**Project name:** Biopython**Project home page:**http://biopython.org/**Operating system:** Platform independent**Programming language:** Python**Other requirements:** Python 2.5 or higher; optional libraries matplotlib, Graphviz, NetworkX**License:** Biopython License**Any restrictions to use by non-academics:** None

## Competing interests

The authors declare that they have no competing interests.

## Authors’ contributions

ET wrote the code for Bio.Phylo. BI wrote the code for Bio.Phylo.PAML. BAC and PJAC mentored and supervised the development of Bio.Phylo and contributed enhancements and bug fixes. ET, BI, PJAC and BAC wrote the manuscript. All authors read and approved the final manuscript.

## Supplementary Material

Additional file 1 Tree rerooting. Example code to read a tree from a file and write copies of the tree rerooted at every internal and external node. The method find_clades produces an iterator over the tree rather than a new list, in order to make more efficient use of memory in general. In this case, because the method root_with_outgroup modifies the tree in-place, which could change the ordering of nodes during traversal, list is used to create an unchanging copy of references to the original nodes in the tree. Click here for file

Additional file 2 Performance benchmark script. Python script to time the execution of the benchmark tasks shown in Table 2. Click here for file
